# Negative pressure pulmonary edema in the prone position: a case report

**DOI:** 10.4076/1757-1626-2-8594

**Published:** 2009-07-27

**Authors:** Hesham Omar, Jaya Kolla, Amrat Anand, Willem Nel, Devanand Mangar, Enrico Camporesi

**Affiliations:** 1 Department of Cardiovascular Medicine, Cairo University Hospital, Cairo, Egypt, Visiting Scholar at Tampa General Hospital, Florida Gulf to Bay Anesthesiology AssociatesPA, Tampa, FloridaUSA; 2 Research Associate, Tampa General Hospital, Florida Gulf to Bay Anesthesiology AssociatesPA, Tampa, FloridaUSA; 3 Florida Gulf to Bay Anesthesiology Associates, PA, Tampa, FloridaUSA; 4 Department of Surgery/Anesthesiology, University of South Florida, Tampa, FL and Florida Gulf to Bay Anesthesiology Associates, PA, Tampa, FloridaUSA

## Abstract

Acute airway obstruction can result in life - threatening pulmonary edema. It can develop rapidly, without warning, in otherwise healthy patients. Negative pressure pulmonary edema has been described after acute airway obstruction in situations when a patient is breathing against an obstructed airway such as croup, epiglottitis or laryngospasm. In the following case, we observed a rare occurrence of pulmonary edema in a female following sedation in the prone position.

## Case presentation

A 49-year-old Caucasian female, with a long history of heavy cigarette smoking and a previous diagnosis of supraventricular tachycardia, for which cardiac ablation was performed 2 years ago, was admitted to the hospital for an elective sacral neuromodulator lead change to treat urinary incontinence. In the operating room, the patient received monitored anesthesia care (MAC) in the prone position. During the procedure she required increasing sedation (IV propofol infusion) and after thirty minutes the patient showed progressive hypercapnia from the nasal cannula sample line, while receiving supplemental oxygen (4 L/min). An oral airway was placed, but end-tidal CO_2_ ranged from 65 to 75 mmHg and did not improve. It was then decided to place a laryngeal mask and to supply positive pressure ventilation; however no improvement of the hypercapnia ensued and we could not provide adequate ventilation. The patient was then emergently intubated to secure an airway and provide adequate ventilation. This required interruption of surgery, turning the patient supine, ventilating oxygen by mask and a succinylcholine bolus. During the intubation process, marked edema above the vocal cords was noticed by an experienced anesthesiologist, allowing only for a smaller sized endotracheal tube to be inserted (6 mm with difficulty, but not a 7 mm tube). The patient was given dexamethasone 8 mg and diphenhydramine 25 mg for the airway edema. Following intubation and after securing the tube in place, we started positive pressure ventilation and returned the patient to the prone position, and the end tidal CO_2_ returned within normal range. However, within a few minutes, the patient developed bibasilar rales, with abundant frothy, blood – tinged secretions appearing in the tube. We maintained the patient prone while the surgical procedure was concluded. The edema fluid was adequately suctioned and abated after 10 minutes of positive pressure ventilation. Postoperatively the patient remained intubated and sedated on a propofol infusion. While in recovery, an arterial blood gas obtained on Synchronized Intermittent Mechanical Ventilation (SIMV) and FiO_2_ of 30%, showed a PO_2_ of 92 mmHg, PCO_2_ of 46 mmHg, pH of 7.34 and HCO_3_ of 25 mEq/L with a SaO_2_ of 97%. Chest X-ray performed after surgery indicated clear lung fields with normal size heart and no evidence of pulmonary edema. The patient’s condition steadily improved and she was subsequently extubated two hours later but continued to be closely monitored overnight and was discharged the following day without requiring an ICU stay.

## Discussion

In this case report we describe a case of NPPE following supraglottic obstruction with deepening sedation in the prone position. Supraglottic obstruction was possibly induced by intense inspiratory effort in a patient who was sedated and hypoventilating leading to airway obstruction and subsequently pulmonary edema. Another possibility is malpositioning (or biting) of the LMA causing inspiratory efforts against an obstructed airway, which could be the cause for the observed supraglottic swelling. The apparent absence of gastric contents in the pulmonary edema fluid, the absence of left ventricular dysfunction in a previously performed echocardiogram and the sequence of the described symptoms make the diagnosis of acute NPPE likely.

NPPE is a relatively uncommon intra-operative and post-operative complication and is still a diagnosis by exclusion of other factors. Generally, NPPE occurs when inspiratory effort against an obstruction creates a large intrapulmonary negative pressure which can increase venous return with subsequent increase in the pulmonary capillary hydrostatic pressure. The transudation of fluid from the pulmonary vasculature causes edema formation into the alveolar space [1,2]. Normal inspiratory pleural pressure ranges from −5 to −8 cm of H_2_O, but in instances of obstruction, inspiratory pressures can increase to −50 cm of H_2_O and in extreme circumstances have been known to exceed −100 cm of H_2_O [3]. Typically, these patients are young athletic males that have well-developed musculature, capable of creating a large negative pressure [4].

NPPE has also been described in detail in children following tonsillectomies. In these patients inspiratory efforts against airway obstruction creates a positive end-expiratory pressure (auto-PEEP) which decreases the trans-capillary pressure gradient (P_c_-P_i_) preventing fluid leak into the interstitium. The sudden relief of the obstruction may lead to loss of the auto-PEEP resulting in a large transcapillary pressure gradient and subsequent pulmonary edema [2,5,6]. NPPE has also been described in patients with upper airway obstruction in cases of epiglottitis and croup, foreign body obstruction, airway secretions, following re-expansion of an atelectatic region of the lung or rapid drainage of a pleural effusion, after near strangulation and after oral and pharyngeal surgery. Other presentations have been described after vigorous suction during bronchoscopy, obstructive sleep apnea and biting on the laryngeal mask tube [2,7,8].

NPPE is not only observed during or after anesthesia; numerous reports in the literature describe situations of respiratory loading requiring large inspiratory efforts, as in submerged scuba divers or breath-holding divers after resurfacing and even swimmers [9,10,11]. The proposed theory behind such a predisposition is that immersion causes central pooling of blood in the pulmonary circulation, either from the peripheral vasoconstrictive effects of cold water, or via increased venous return as caused by warm water immersion. Ultimately, intense inspiratory efforts while immersed in water may create a large negative intrathoracic pressure. The combination of these factors leads to stress failure of pulmonary capillaries and the breakdown of the alveolar wall, causing high-permeability’ pulmonary edema or even frank hemorrhage [12].

Treatment of NPPE is usually supportive and includes maintenance of a patent airway and adequate oxygenation. The uses of dexamethasone and diuretics have not shown to be efficacious in prospective studies and may be deleterious [7]. In severe cases endotracheal intubation and mechanical ventilation with PEEP is usually necessary. The prognosis is usually benign, but 11% to 40% of the described cases develop life threatening complications, including mortality [2].

In conclusion, negative pressure pulmonary edema is a possible cause of respiratory distress in the operating room, especially in patients breathing spontaneously while in the prone position. Any compromise of the airway in the prone position is difficult to recover. We therefore suggest that these patients receive adequate protection of their airway, comprising aggressive intubation, general anesthesia and continuous ventilatory support.

## Abbreviations

LMA: Laryngeal mask airway
NPPE: Negative pressure pulmonary edema
PEEP: Positive end expiratory pressure
P_c_: Capillary hydrostatic pressure
P_i_: Interstitial pressure
